# Translation and validation of the Child Three-Factor Eating Questionnaire (CTFEQr17) in French-speaking Canadian children and adolescents

**DOI:** 10.1017/S136898002100392X

**Published:** 2022-03

**Authors:** Isabelle Frappier, Raphaëlle Jacob, Shirin Panahi, David Larose, Eleanor J Bryant, Jean-Philippe Chaput, David Thivel, Vicky Drapeau

**Affiliations:** 1 Centre NUTRISS, Institute of Nutrition and Functional Foods, Université Laval, Québec City, QC, Canada; 2 School of Nutrition, Université Laval, Québec City, QC, Canada; 3 Québec Heart and Lung Institute Research Centre, Université Laval, Québec City, QC, Canada; 4 Centre de Recherche Interuniversitaire sur la Formation et la Profession Enseignante (CRIFPE), Université Laval, Québec City, QC, Canada; 5 Department of Physical Education, Université Laval, 2300, Rue de la Terrasse, Québec City, QC, Canada G1V 0A6; 6 Department of Kinesiology, Université Laval, Québec City, QC, Canada; 7 Faculty of Social Sciences, University of Bradford, Bradford, UK; 8 Healthy Active Living and Obesity Research Group, Children’s Hospital of Eastern Ontario Research Institute, Ottawa, Canada; 9 Laboratory of the Metabolic Adaptations to Exercise under Physiological and Pathological Conditions, Clermont Auvergne University, Clermont-Ferrand, France

**Keywords:** Obesity, Children, Eating behaviours, Diet quality, Validation, Three-factor eating questionnaire

## Abstract

**Objective:**

To translate and validate the Child Three-Factor Eating Questionnaire (CTFEQr17), assessing cognitive restraint (CR), uncontrolled eating (UE) and emotional eating (EE), among French-speaking Canadian young individuals.

**Design:**

Phase 1 comprised a translation and the evaluation of the comprehension of the questionnaire. Phase 2 comprised a confirmatory factor analysis (CFA), the evaluation of internal consistency (Cronbach’s *α*), test–retest reliability (intra-class correlation coefficients (ICC)) and construct validity, including correlations among the CTFEQr17 and Eating Attitudes Test (EAT-26), anthropometrics, dietary intake and diet quality.

**Setting:**

Primary and secondary schools, Québec City, Canada.

**Participants:**

Phases 1 and 2 included 20 (40 % boys, mean age 11·5 (sd 2·4) years) and 145 (48 % boys, mean age 11·0 (sd 1·9) years) participants, respectively.

**Results:**

Phase 1 resulted in the questionnaire to be used in Phase 2. In Phase 2, the CFA revealed that the seventeen item, three-factor model (CTFEQr17) provided an excellent fit. Internal consistency was good (Cronbach’s *α*: 0·81–0·90). Test–retest reliability was moderate to good (ICC = 0·59, (95 % CI 0·48, 0·70), ICC = 0·78, (95 % CI 0·70, 0·84), ICC = 0·50, (95 % CI 0·38, 0·62) for CR, UE and EE, respectively). CR correlated with EAT-26 score (*r* = 0·43, *P* < 0·0001). UE and EE correlated negatively with BMI *Z*-scores (*r* = −0;·26, *P* = 0·003; *r* = −0;·19, *P* = 0·03, respectively). CR correlated with the proportion of energy intake from protein and diet quality (*r* = 0·18, *P* = 0·04; *r* = 0·20, *P* = 0·02, respectively).

**Conclusion:**

The CTFEQr17 is suitable to use among French-speaking Canadian young individuals.

In Canada, overweight and obesity affect nearly one in three children^(1)^. Since children with obesity are at greater risk of obesity and related complications in adulthood^(2–5)^, it is important to focus on strategies to prevent long-term consequences associated with body weight gain^(3,4)^. While the causes of childhood obesity are complex^(6,7)^, eating behaviour traits have been suggested to influence body weight and obesity among adults^(8)^. Moreover, eating behaviours have been shown to partially mediate genetic susceptibility to obesity in adults^(9)^. Among eating behaviours, disinhibition (i.e. overconsumption of food in response to cognitive or emotional cues) and susceptibility to hunger (i.e. food intake in response to feelings and perceptions of hunger) have been correlated with a greater risk of overweight and obesity in adults in the Québec Family Study^(10)^. High cognitive restraint (CR) level (i.e. conscious efforts to limit food intake to control or lose body weight) was also correlated with greater weight gain after 6 years of follow-up^(11)^. In children, eating behaviours have been shown to predict changes in weight status over time^(12)^. In adolescents with obesity, CR has been correlated with greater *ad libitum* energy intake (EI) following a weight loss program, potentially favouring weight regain^(13)^.

In adults, eating behaviours are widely assessed with the Three-Factor Eating Questionnaire (TFEQ)^(14)^ that measures three dimensions of eating behaviours: CR, disinhibition and susceptibility to hunger^(14)^. Two shortened versions of this questionnaire were subsequently developed, resulting in the TFEQ-R18^(15)^ and TFEQ-R21^(16)^. Both versions include the measurement of CR which was retained from the original TFEQ, uncontrolled eating (UE) (i.e. tendency to overeat and eat in response to multiple stimuli) which results in the combination of disinhibition and hunger factors from the original TFEQ, and emotional eating (EE) (i.e. eating in response to negative emotions) which emerges from some disinhibition items^(15,16)^. The TFEQ-R21 questionnaire was later validated in an adult population and refined, resulting in the TFEQ-R18V2^(17)^. Although this latter study found only small correlations with eating behaviour traits and BMI^(17)^, other studies in adult populations have shown that UE^(18,19)^, EE and CR^(19,20)^ were correlated with a higher BMI. The same correlations have been found in a sample of young females^(21)^. In children populations, eating behaviour traits have also been correlated with BMI and BMI *Z*-score^(22–27)^ and food preferences^(25,26)^. However, little is known about the relationship between eating behaviours, obesity and diet in children and adolescents.

To better understand the importance of eating behaviour traits in relation to childhood obesity, researchers validated an English^(25)^ and Spanish^(23)^ version of the TFEQ-R21 adapted for children and adolescents. The validation study conducted by Bryant and colleagues resulted in a seventeen-item version of the questionnaire (CTFEQr17) and reported good internal consistency^(25)^. This questionnaire was also later validated in an English-Canadian sample, revealing a four-factor and twenty-item structure^(26)^, a Romanian sample, preserving the twenty-one-item original structure^(24)^ and a Turkish sample, resulting in the same seventeen-item version as validated by Bryant and colleagues^(27)^. While it could represent an essential aspect of improving the treatment and prevention of childhood obesity, there is currently no validated French version of the CTFEQ.

This study aimed to translate and validate a French version of the Child Three-Factor Eating Questionnaire in a sample of French-speaking Canadian children and adolescents and also aimed to examine internal consistency reliability, test–retest reliability and construct validity with the correlations among eating behaviours and eating attitudes, anthropometric measures, dietary intake and diet quality. Regarding construct validity, we hypothesise that CR will be positively correlated with Eating Attitudes Test (EAT-26) score and negatively correlated with EI, waist circumference (WC) and BMI *Z*-score, and that UE and EE will be positively correlated with EI, WC and BMI *Z*-score.

## Methods

### Phase 1: Translation of the CTFEQ and evaluation of the comprehension and acceptability of the questionnaire

A French translation of the twenty-one-item CTFEQ developed by Bryant and colleagues^(25)^ was conducted and then back-translated into English by one individual with proficiency in both English and French and by a certified translator. Both English versions were compared, and the French version was adjusted to obtain a final French version. To ensure its understandability for children and adolescents, the French translation was evaluated by a child nutrition expert and by one paediatrician/researcher. Informal verification of the understanding of the questionnaire was conducted in a sample of three young individuals aged between 8 and 15 years, who provided verbal comments about their understanding of the questionnaire. Formal understanding and acceptability of the questionnaire were then assessed within a small group of young individuals recruited via an informal network of the research team (*n* 20 participants aged 8–15 years; mean age 11·5 (sd 2·4) years), through semi-structured interviews that were about 20 min in duration. Participants were asked to complete the questionnaire, to comment on each item and scale and to rephrase the instructions to identify misunderstood words or items. The interviewer completed an evaluation sheet identifying the elements, concepts or words that were not understood by the participants. The mean duration time of the questionnaire completion was 4·7 (sd 1·1) minutes. All items and scales were judged to be well understood by the participants; therefore, no item was modified or removed. This phase resulted in a final French version of the CTFEQ that was used in Phase 2.

### Phase 2: Validation of the CTFEQ

#### Participants

Participants included 162 children and adolescents recruited from four primary and secondary schools in Québec City, Canada. School recruitment was conducted by contacting school principals by email. Participant recruitment was conducted via personal contact. Inclusion criteria were being aged between 8 and 15 years, francophone and having no significant learning difficulties (e.g. dyslexia), which was screened by teachers at schools before recruitment for participants. A total of 17 participants did not complete the study because they were absent from school during the data collection period. Thus, the final sample comprised of 145 individuals.

#### Study protocol

**Fig. 1:**
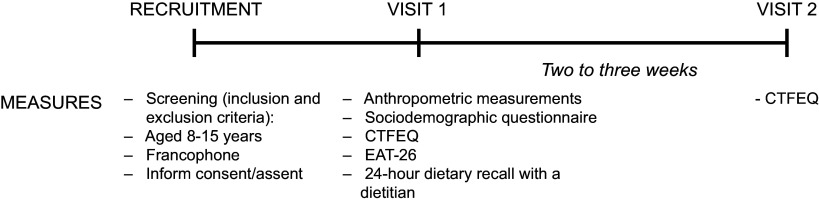
Study protocol

This study included two visits taking place in schools. During the first visit, anthropometric measurements were performed. Then, each participant completed questionnaires including the CTFEQ, and a 24-h dietary recall administered by a registered dietitian. Two to three weeks after the first visit, a second visit took place in schools to complete the CTFEQ a second time (Fig. 1).

### Measures

#### Sociodemographic characteristics

A sociodemographic questionnaire was completed during the first visit to obtain information on the date of birth, sex, school grade and ethnicity of each participant.

#### Child Three-Factor Eating Questionnaire (CTFEQ)

The CTFEQ is a twenty-one-item self-reported questionnaire developed by Bryant and colleagues^(25)^, based on the Three-Factor Eating Questionnaire-R21 (TFEQ-R21)^(14)^. This questionnaire assesses CR, a six-item factor, UE, a nine-item factor and EE, a six-item factor. Items 1–20 are measured on a four-point Likert scale and item 21 is measured on an eight-point Likert scale, which is then converted into a four-point scale. Mean scores for each factor are calculated with the same coding procedure as presented by Bryant and colleagues^(25)^: items 1–16 were reverse coded, items 17–20 were normally coded and item 21 was modulated into a four-point scale (1–2 = 1; 3–4 = 2; 5–6 = 3; 7–8 = 4) (see online Appendix in Supplemental Material).

#### Eating Attitudes Test (EAT-26)

The French validated version of the EAT-26^(28)^, which is a self-administrated questionnaire, was administered. This questionnaire measures symptoms associated with eating disorders, including dieting (i.e. avoidance of fattening foods and preoccupation with being thinner), bulimia and food preoccupation (i.e. thoughts about food and thoughts indicating bulimia) and oral control (i.e. self-control of eating and perceived pressure from others to gain weight)^(29)^. Participants obtaining a total score of over 20 are suggested to be at risk of having an eating disorder^(29)^.

#### Anthropometrics

Body weight was measured with light clothing and without shoes using a portable bioimpedance weighing scale (TANITA, model TBF-310) and recorded to the nearest 0·1 kg. Height was obtained using a portable stadiometer and recorded to the nearest 0·1 cm. WC was measured midway between the lowest rib and the top of the iliac crest with a measuring tape to the nearest 0·1 cm. Two WC measures were taken, and the average was used in analyses. BMI was calculated as the weight (kg) divided by height squared (m^2^) and then converted into a BMI *Z*-score^(30)^. BMI *Z*-score was calculated according to the WHO procedure^(31)^, using the WHO anthropometric *Z*-scores 0–19 years calculator^(32)^. Children were then classified into four weight categories as follows: underweight (BMI *Z*-score < −2), normal weight (1 > BMI *Z*-score ≥ −2), overweight (2 > BMI *Z*-score ≥ 1) or obese (BMI *Z*-score ≥ 2).

#### Dietary intake and diet quality

One 24-h dietary recall was completed for each participant during the first visit by three registered dietitians. Detailed information on all foods and beverages consumed during the previous day was collected using the USDA five-step multiple-pass method^(33,34)^. To increase the accuracy of food portion estimations, food models and standard kitchen measures were used. Dietary recalls were analysed with Nutrific nutrient analysis software version 2015 (Université Laval, Québec, QC, Canada) which is based on the Canadian Nutrient File version 2015^(35)^, to obtain energy, macronutrient and micronutrient intakes. Two 24-h dietary recalls were excluded from the analysis due to the lack of information provided by the participants during the interviews. Diet quality was assessed using the Nutrient-Rich Food Index 9·3 (NRF 9.3), a nutritional quality index based on nine nutrients to encourage (i.e. protein, fibre, vitamins A, E and C, calcium, iron, potassium and magnesium) and three nutrients to limit (i.e. SFA, total sugars and sodium)^(36,37)^. A higher Nutrient-Rich Food Index 9·3 score indicates a higher diet quality.

### Statistical analysis

The complete database was examined for missing data. For each questionnaire, imputation was performed when there was a maximum of two missing items per factor per participant. Imputation procedure consisted in replacing the missing data with the mean value of its associated item. Mean data imputation was performed since it is an acceptable imputation method when proportions of missing data reach up to 10 %^(38)^ and that only small proportions of missing data were found in this study. For the CTFEQ questionnaire completed at the first and second visits, 1·3 % and 0·6 % of the data were missing, resulting in 25·0 % and 92·9 % of the missing data being imputed, respectively. For the EAT-26 questionnaire, 1·5 % of data were missing and 39·3 % were imputed. Prior to the analyses, distributions of the quantitative variables were evaluated based on the skewness and kurtosis indices and by visual inspection. Data were also examined for outliers with the JMP Outlier Analysis report. One outlier for the EAT-26 questionnaire was removed and two outliers for nutritional data were also removed for all statistical analysis.

#### Descriptive analysis

A descriptive analysis for participant characteristics was performed. Sex differences in participant characteristics were assessed using Student *t*-tests for continuous variables and *χ* ^2^ tests for nominal variables.

#### Factorial structure

Confirmatory factor analysis using the maximum likelihood method with robust options for ordinal categorical variables was performed. The fit to the data was tested on three different models of the CTFEQ found in the literature to determine the most suitable factorial structure. Model 1 included the twenty-one-item model loading into three factors validated by Martín-García *et al.* ^(23)^ and later validated by Steff *et al.* ^(24)^. Model 2 included the seventeen-item model loading into three factors validated by Bryant *et al.* ^(25)^. Model 3 included the twenty-item model loading into four factors validated by Yabsley *et al.* ^(26)^. The adequacy of the model fit was determined according to the different fit indices resulting from each factor analysis, using the thresholds proposed by Hu and Bentler^(39)^. A Non-Normed Fit Index > 0·95; a Comparative Fit Index > 0·95 and a Root Mean Squared Error of Approximation ≤ 0·06 were considered adequate^(39)^. *χ* ^2^ and normed chi-square values (*χ* ^2^/df) were assessed, since regular *χ* ^2^ values tend to be affected by sample size^(40)^. Values less than 5 and 2 indicate an acceptable and very good fit, respectively^(41,42)^. Standardised factor loadings were assessed in each confirmatory factor analysis. Akaike’s Information Criteria (AIC) was also assessed, with smaller values of AIC indicating the most parsimonious model^(43)^.

#### Reliability

Cronbach’s *α* coefficients based on polychoric correlations were used to evaluate internal consistency reliability for each factor of the models tested (i.e. adequate internal consistency as Cronbach’s *α* ≥ 0·70)^(42,43)^. Intra-class correlation coefficients (ICC) were assessed with the Macro % ICC9 for SAS two-way mixed-effects^(44)^ for each factor using data from the two completions of the questionnaire to determine test–retest reliability. The 95 % CI for each ICC was used to determine the level of reliability^(45)^. Values less than 0·50 show poor reliability, values between 0·50 and 0·75 show moderate reliability, values between 0·75 and 0·90 show good reliability and values above 0·90 show excellent reliability^(45)^.

#### Construct validity

Pearson’s correlations were performed between the CTFEQr17 scores and age for boys and girls independently since sex was a significant covariate for CR and EE. Pearson’s partial correlations were performed between the CTFEQr17 scores for each factor and eating attitudes, anthropometric measures, dietary intake and diet quality. All correlations were adjusted for sex and age. Interaction for sex, age and body weight status were assessed. Correlations assessing dietary intakes and diet quality were performed without and with adjustments for misreporting. Subjects that were under-reporters and over-reporters were identified using the calculating method suggested by Huang *et al.*, taking into account reported EI and predicted total energy expenditure (TEE)^(46)^. Predicted TEE was assessed with the Institute of Medicine’s equations for dietary reference intake^(47)^. Since this study did not evaluate the objective measurement of physical activity level, subjects were assigned with low active physical activity coefficient of 1·4, as proposed by Huang *et al.* ^(46)^, which is supported by the fact that only 39 % of Canadian children and youth meet the required physical activity recommendations^(48)^. Participants were classified as under, over and plausible reporters when the % EI/TEE was lower, higher or within the 1 sd cutoff, respectively, for the following equation: 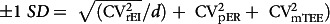 , in which CV_rEI_ is the intra-variation of EI (%), where a value of 23 %^(49)^ was used; *d* is the number of recording days (1 in the present study); CV_pER_ is the error of the parameters in the equations of predicted energy requirements, including physical activity level, where a value of 15 % was obtained; and CV_mTEE_ is the day-to-day variation and measurement error for TEE based on doubly labelled water technique, where a value of 8·2 % was used^(46)^. The cutoffs were exponentiated since EI distribution is skewed. Confirmatory factor analysis and Cronbach’s *α* coefficients calculation were performed on EQS Multivariate Software, version 6.2. All the remaining analyses were performed on JMP software, version 14.0 from Statistical Analysis System (SAS Institute) and SAS software, version 9.4 (SAS Institute). Results are presented as mean (sd) or sd. Statistical significance was set at *P* < 0·05.

#### Sample size

A minimum of 100 participants was necessary to guarantee the stability of the variance–covariance matrix in confirmatory analysis^(50)^. The number of participants needed for confirmatory factor analysis of the three-factor and twenty-one-item model was calculated and set to 119 participants required for a statistical power level of 80 %, a significance level of 0·05 and an anticipated effect size of 0·3^(51)^. This sample size was also similar to previous CTFEQ validation studies (Steff *et al.*, *n* 153^(24)^; Yabsley *et al.*, *n* 158^(26)^). However, to consider missing data or absences from school at the second data collection, we planned to recruit about 160–165 participants.

## Results

### Descriptive analysis

The mean age of participants was 11·0 (sd 1·9) years (Table 1). The majority of the participants were girls and Caucasians. The mean BMI *Z*-score was 0·03 (sd 0·94) and was significantly different for girls in comparison to boys (*P* = 0·01).

**Table 1:** Participant characteristics

Variable	Total (*n* = 145)		Boys (*n* = 69)		Girls (*n* = 76)		*P* *
	*n*	%	*n*	%	*n*	%	
Age (years)†							
Mean	11·0		10·8		11·3		0·13
sd	1·9		1·9		1·9		
Anthropometric measurements							
Weight (kg)‡							
Mean	37·1		36·4		37·5		0·53
sd	10·0		10·1		10·0		
BMI *Z*-score§							
Mean	0·03		0·25		−0·16		0·01
sd	0·94		0·92		0·93		
WC (cm)‡							
Mean	62·8		63·1		62·4		0·57
sd	7·0		7·1		7·0		
School grade							
Primary school, grades 3–4 (8–10 years)	60	41·4	32	46·4	28	36·8	0·06
Primary school, grades 5–6 (10–12 years)	53	36·6	24	34·8	29	38·2	
Middle school, grades 7–9 (12–15 years)	29	20·0	10	14·5	19	25·0	
Other||	3	2·1	3	4·3	0	0	
Ethnicity							
Caucasian	127	87·6	58	84·1	69	90·8	0·22
Other¶	18	12·4	11	15·9	7	9·2	

BMI, Body Mass Index; WC, waist circumference.

* *P* values indicate gender differences.

† *n* 143.

‡ *n* 142.

§ *n* 138.

|| Particular path.

¶ For boys, *n* 0 First Nation, *n* 3 Asians, *n* 2 Afro-Canadians, *n* 1 Latino-Canadian, *n* 5 other ethnicity. For girls, *n* 1 First Nation, *n* 2 Asians, *n* 1 Afro-Canadian, *n* 0 Latino-Canadian, *n* 3 other ethnicity.

Data are presented means (sd) or otherwise specified.

### Factorial structure

All three models tested in confirmatory factor analysis had an excellent fit to the data, meeting the recommended thresholds for each fit index as indicated by Non-Normed Fit Index and Comparative Fit Index above 0·95, Root Mean Squared Error of Approximation below 0·06 and *χ* ^2^/df below 2 (Table 2). Model 3 demonstrated a weaker fit to the data compared with the two other models and therefore was not retained. Model 1 had a lower AIC value (−195·38) than Model 2 (−126·05). No weak items for all three models were identified, with all standardised factor loadings being above 0·3 (see Table 3 for Model 2, results not shown for Models 1 and 3). However, Models 1 and 3 had one weaker factor loading under 0·4 (items 17 and 18, respectively), while all factor loadings of Model 2 were greater than 0·4.

**Table 2:** Fit indices for the three models of the CTFEQ evaluated by CFA

Model	Items	Factors	*χ* ^2^ (df)	*χ* ^2^/df	NNFI	CFI	RMSEA	90 % CI	AIC
Model 1 (Martín-García *et al.*, 2016)	21	3	176·621 (186)(*P* = 0·7)	0·95	1·004	1·000	0·000	0·000, 0·031	−195·379
Model 2 (Bryant *et al.*, 2018)	17	3	105·950 (116)(*P* = 0·7)	0·91	1·005	1·000	0·000	0·000, 0·032	−126·050
Model 3 (Yabsley *et al.*, 2018)	20	4	190·551 (164)(*P* = 0·1)	1·16	0·957	0·963	0·034	0·000, 0·053	−137·449

CFA, confirmatory factor analysis; df, degrees of freedom; NNFI, Non-Normed Fit Index; CFI, Comparative Fit Index; RMSEA, Root Mean Squared Error of Approximation; AIC, Akaike’s Information Criteria.

*n* 142.

**Table 3:** Factor loadings evaluated by CFA, internal consistency (Cronbach’s *α*), test–retest reliability (intra-class correlation coefficients) and descriptive statistics (mean ± sd) for the retained CTFEQr17

Factors	Items	Factor loadings	Cronbach’s *α*	Test–retest ICC	95 % CI	Total (*n* = 145)		Boys (*n* = 69)		Girls (*n* = 76)		*P* *
						Mean	sd	Mean	sd	Mean	sd	
Cognitive restraint	1	0·683	0·81	0·59	0·48, 0·70	1·8	0·7	1·9†	0·7	1·7†	0·7	0·04
	5	0·646										
	11	0·965										
Uncontrolled eating	3	0·816	0·88	0·78	0·70, 0·84	2·3	0·7	2·2	0·7	2·4	0·7	0·1
	6	0·703										
	8	0·766										
	9	0·516										
	12	0·750										
	13	0·680										
	15	0·785										
	20	0·489										
Emotional eating	2	0·689	0·90	0·50	0·38, 0·62	1·4	0·5	1·3‡	0·4	1·5‡	0·6	0·03
	4	0·714										
	7	0·825										
	10	0·796										
	14	0·825										
	16	0·808										

CFA, confirmatory factor analysis; ICC, intra-class correlation coefficient.

* *P* values indicate gender differences.

† Boys had significantly higher CR than girls.

‡ Girls had significantly higher EE than boys.

Student’s *t* test for differences between boys and girls.

*n* 145.

### Reliability

For Model 1, Cronbach’s *α* coefficients were all adequate (0·78, 0·88 and 0·90 for CR, UE and EE, respectively). In Model 2, the Cronbach’s *α* coefficient for CR increased from 0·78 to 0·81 following the removal of items 17, 18 and 21 (Table 3). The coefficients for UE, in which item 19 was removed, and EE both remained the same (Table 3). Test–retest reliability for Model 1 was poor to moderate for CR and EE (ICC = 0·48; (95 % CI 0·35, 0·61) and ICC = 0·50; (95 % CI 0·38, 0·62), respectively) and moderate to good for UE (ICC = 0·77; 95 % CI 0·70, 0·84)). ICC for both CR and UE increased in Model 2 (ICC = 0·59; (95 % CI 0·48, 0·70) and ICC = 0·78; (95 % CI 0·70, 0·84), respectively) and remained the same for EE (Table 3), therefore demonstrating a superior test–retest reliability for the seventeen-item model compared with the twenty-one-item model (Model 1). Thus, the Model 2, comprising seventeen items and three factors as validated by Bryant and colleagues (CTFEQr17)^(25)^, was selected since it was offering an excellent model fit and a superior internal reliability and test–retest reliability compared to Model 1, especially for the CR factor.

### Construct validity

Boys had slightly but significantly higher CR than girls (*P* = 0·04) and girls had slightly but significantly higher EE than boys (*P* = 0·03) (Table 3). CR correlated negatively with age for boys and girls (*r* = −0·37, *P* = 0·002; *r* = −0·24, *P* = 0·04, respectively). UE correlated negatively with age for girls only (*r* = -0·34, *P* = 0·003). No correlation between EE and age, separated by sex, was found.

As presented in Table 4, there was a positive correlation between UE and EE (*r* = 0·61, *P* < 0·0001), but neither UE nor EE correlated with CR. CR was the only factor to correlate significantly with EAT-26 score (*r* = 0·43, *P* < 0·0001). UE and EE correlated negatively with BMI *Z*-score (*r* = −0·26, *P* = 0·003 and *r* = −0;·19, *P* = 0·03, respectively). Only UE correlated negatively with WC (*r* = −0·21, *P* = 0·01) even though a tendency towards a negative correlation with WC can be observed with EE (*r* = −0·16, *P* = 0·07). CR correlated positively with the percentage of EI from protein (*r* = 0·18, *P* = 0·04) and with Nutrient-Rich Food Index 9·3 (*r* = 0·20, *P* = 0·02) before adjustments were made for misreporting. After adjustments for misreporting, correlation with dietary intakes and diet quality did not change. For both methods, a trend towards a negative correlation between CR and EI was obtained (*r* = −0·16, *P* = 0·07 and *r* = −0·17, *P* = 0·06, without and with adjustments for misreporting, respectively).

**Table 4:** Correlations between the retained CTFEQr17 factors and eating attitudes, anthropometrics, dietary intake and diet quality*

Variable	Cognitive restraint		Uncontrolled eating		Emotional eating	
	*r*	*P*	*r*	*P*	*r*	*P*
Eating behaviours						
Cognitive restraint	1					
Uncontrolled eating	0·10	0·2	1			
Emotional eating	0·14	0·1	0·61	<0·0001	1	
EAT-26	0·43	<0·0001	0·13	0·1	0·14	0·1
Anthropometric measurements						
BMI *Z*-score	0·07	0·4	−0·26	0·003	−0·19	0·03
WC, cm	0·05	0·6	−0·21	0·01	−0·16	0·07
Dietary intake and diet quality						
Energy intake, kcal	−0·16	0·07	−0·05	0·5	−0·09	0·3
% carbohydrates	0·01	0·95	0·00	0·99	−0·01	0·9
% protein	0·18	0·04	−0·01	0·9	−0·05	0·6
% lipids	−0·08	0·3	−0·00	0·96	0·03	0·7
NRF 9·3	0·20	0·02	−0·11	0·2	−0·03	0·7
Dietary intake and diet quality†						
Energy intake, kcal†	−0·17	0·06	−0·07	0·5	0·01	0·9
% carbohydrates†	−0·01	0·9	0·03	0·8	−0·02	0·8
% protein†	0·19	0·03	−0·02	0·8	−0·05	0·6
% lipids†	−0·08	0·4	−0·03	0·7	0·04	0·7
NRF 9·3†	0·18	0·04	−0·12	0·2	−0·05	0·5

EAT-26, Eating Attitudes Test; BMI, Body Mass Index; WC, waist circumference; NRF 9.3, Nutrient-Rich Foods Index 9.3.

* Correlations determined with partial Pearson’s correlation coefficients adjusted for sex and age.

† Values adjusted for misreporting.

## Discussion

The main objective of this study was to produce and validate the French version of the Child Three-Factor Eating Questionnaire^(25)^ in a French-speaking sample of Canadian children and adolescents aged 8–15 years. Results suggest that the French version of the seventeen-item model CTFEQ (CTFEQr17) confers an excellent factor structure and internal consistency, as well as moderate to adequate test–retest reliability in young French-speaking children and adolescents. As expected, CR correlated to EAT-26 score, but not with EI or WC and BMI *Z*-score thus confirming partly our hypothesis. It was also correlated with the proportion of EI from protein and diet quality. UE and EE correlated negatively only with BMI *Z*-score, the opposite of what was expected.

### Factorial structure and reliability

The present study demonstrated that the model including seventeen items and three factors (CTFEQr17) had a superior model fit in a sample of French-speaking Canadian children and adolescents compared to other models found in the literature (twenty items, four factors and twenty-one items, three factors)^(23,24,26)^, therefore reproducing the results obtained by Bryant and colleagues^(25)^. CR factor showed weaker internal consistency in comparison to UE and EE. This has also been shown in the Spanish^(23)^, English^(25)^, Romanian^(24)^ and Turkish^(27)^ versions of the CTFEQ. In this study, both internal consistency and test–retest reliability of the questionnaire increased after the removal of items 17, 18 and 21 of the CR factor (Model 2), suggesting that some items in this factor may be less understood by the children. In other studies, items 17, 18^(25)^ or 21^(25,26)^ of the CR factor have also been removed from the original factor structure due to weaker fit, thus improving validity^(25,26)^. However, the Spanish^(23)^ and Romanian^(24)^ versions of the CTFEQ found a better fit with the complete questionnaire, retaining all six items comprising CR^(23,24)^. In adult populations, items 17, 18 and 21 have also been shown to be problematic in one previous validation study among Canadian and American populations^(17)^, but most studies demonstrated a strong CR factor, retaining its six items^(15,16,21)^. One study among French adults and adolescents observed a weaker CR factor, but still preserved it^(52)^, while other studies among adolescents showed a weaker internal consistency of the CR factor compared to UE and EE factors^(53,54)^. These results suggest that the six-item CR factor could be weaker in comparison to UE and EE, but this factor was judged to be adequate in the present study after the removal of items 17, 18 and 21, as in the model proposed by Bryant *et al.* ^(25)^.

### Construct validity

The results of this study suggest that eating behaviour traits among children and adolescents may be influenced by sex and age. Boys had a higher CR than girls. However, previous studies have shown the opposite result^(52,55)^. This study also showed that girls reported a higher EE than boys, which is supported by other studies^(52,55,56)^. This may indicate that girls respond more to emotions to influence their eating habits compared to boys. Other studies did not demonstrate any differences between sexes at this age^(23,26)^, while some observed a significant difference for UE^(25,52)^, which highlights possible differences in samples used related to sex. According to Cohen’s effect size^(57)^, the strength of these correlations represented a small effect size for CR (*r* = 0·29) and medium effect size for EE (*r* = 0·39), indicating that these correlations might not be clinically meaningful. Age may also be involved in influencing eating behaviours. Accordingly, in this study, CR was negatively correlated with age in boys and girls and UE was negatively correlated with age in girls only. These correlations are supported by other studies among children and adolescents, for CR^(25,26)^ and UE^(23,25,26)^. Younger individuals may be sensitive to parental eating practices and children’s eating behaviours are influenced by the parent’s own eating behaviours^(58)^. Therefore, younger individuals might be more sensitive to their parent’s pressures to control their food intake which may influence their eating behaviours, like CR, while adolescents might be less likely to be under the direct influence of their parents regarding their eating behaviours. Our results also show that younger individuals may be more sensitive to internal or environmental stimuli about how they eat. As in this study, no correlation between age and EE was observed in previous studies^(23,25,26)^.

The present study shows a moderate positive correlation between UE and EE. The CR factor did not significantly correlate with any other factor. Both of these results are in line with the English and Romanian versions of the questionnaire^(24,25)^ but contradictory to the Spanish version, which demonstrated a negative correlation between UE and CR^(23)^ and the English version in a Canadian sample, which showed a positive correlation between EE and CR^(26)^. However, both of these versions also observed a positive correlation between UE and EE^(23,26)^. As UE and EE derive from disinhibition and susceptibility to hunger in the original TFEQ and imply overeating, this correlation is therefore expected. While restraint has been known to be correlated with overeating^(59)^, it has also been previously shown that disinhibition can be present independently of restraint^(60)^, therefore supporting the lack of correlation between CR and UE or EE in this study. The disparity in results for CR correlations with other eating behaviours may be the result of cultural or social differences in the samples studied. Further studies are needed to understand the relationship between CR and overeating in children populations.

This study observed a moderate positive correlation between CR and EAT-26, which characterises the presence of symptoms associated with eating disorders. Compared to non-restrained eaters, restrained eaters are more likely to express disordered eating patterns^(61)^. Previous studies have also shown positive correlations between disordered eating attitudes (EAT-26) and restrained eating^(62–65)^, which supports these results.

The negative correlations between both UE and EE factors and BMI *Z*-scores and between UE and WC are supported by a previous study demonstrating an inverse correlation between UE and EE and BMI *Z*-scores, but only in boys^(26)^, and other studies demonstrating a negative correlation between UE and BMI^(23)^ and UE being significantly lower among children and adolescents with obesity or overweight compared to those of normal weight^(27)^. In contrast to those results, positive correlations have been shown between both UE and EE and body weight and BMI^(24)^. Among adolescents, EE and UE have also been shown to be positively correlated with BMI, assessed with the adult TFEQ-R18^(21,53)^, while disinhibition has been positively correlated with BMI in children^(22,66)^ and adolescents^(67–69)^. Overall, these results may imply that the young normal weight individuals reporting higher UE and EE are at higher risk for future weight gain. Longitudinal studies are needed in children to investigate these correlations. To help better understand these results, future studies assessing the CTFEQr17 in relation to eating behaviours and anthropometric measures should consider a larger sample size and life stage (e.g. childhood, adolescence, adulthood).

Currently, no studies among children and adolescents have evaluated the correlations between dietary intake, diet quality and eating behaviour traits as measured with the CTFEQ. In this study, CR was positively correlated with the proportion of energy derived from protein and diet quality, both with and without adjustments for misreporting. Among female teenagers and young adults^(52)^ and children and adults^(56)^, a higher proportion of energy derived from protein was also correlated with CR, but both studies also observed a negative correlation between EI and CR^(52,56)^, which was not shown in the present study although a tendency towards this correlation was obtained. Another study among young girls observed that at ages 7 and 9 years, restraint was negatively correlated with EI^(70)^. It could be possible that the single 24-h dietary recall used in this study may not be enough to observe under-consumption since correlations between CR and lower EI were NS. With regard to diet quality, a negative correlation between CR and the consumption of energy-dense foods was observed among teenagers and young adults^(52)^ and a positive correlation between restraint and both the consumption of fruits and vegetables and eating fast food less frequently was observed among young adult men^(71)^, which supports our results. This suggests that restrained individuals tend to choose foods of higher nutritional quality and richer in protein, perhaps to influence their body weight and their satiety. Regarding UE and EE, they have been previously positively correlated with EI^(52)^. Other studies observed that children with high UE had a greater preference for high-fat foods^(25,26)^. The present study did not observe any correlation between dietary intakes and UE or EE, perhaps due to its single measured observation of food intake.

### Strengths and limitations

A strength of this study was the evaluation of test–retest reliability, a measurement that allows the adequate assessment of temporal stability of the results obtained by the same person. Also, an assessment of children’s dietary intakes rather than food preferences was included, in contrast to previous studies^(25,26)^. The 24-h dietary recall allowed the acquirement of more precise information on the actual intakes of the participants. One limitation of this study is that the pubertal stage via Tanner staging was not evaluated, which has been shown to influence eating behaviour traits in previous work^(23)^. Furthermore, information on the socioeconomic level of participants was not collected which may also influence study outcomes since correlations between eating behaviour traits and socioeconomic level have previously been shown among adolescents^(55)^ and adults^(72–74)^. Additionally, although the 24-h dietary recall is a well-established method to assess children’s dietary intake^(75)^, the present study only collected one 24-h dietary recall during the first visit. Usually, repeated recalls should be collected to assess the population distribution of usual intakes^(76)^ and many studies use repeated 24-h dietary recall instead of a single observation^(77)^. As with any dietary assessment method, individuals with overweight and obesity^(75–81)^, who engage in higher screen time or higher moderate to vigorous physical activity^(79)^, who have higher sedentary behavior or lower socio-economic status^(81)^ and adolescent girls^(77)^ may misreport their food intake. Under-reporting of dietary intakes has also been shown to increase with age among children^(77)^ and to be higher among restrained adolescents^(79)^, which justifies adjustments for misreporting. However, it is important to acknowledge the fact that the adjustment method assumes weight stability^(46)^ and does not distinguish individuals who truly under-eat or over-eat from those who are under-reporting or over-reporting their intakes which may bias the results^(79,81)^.

## Conclusion

The CTFEQr17 is a valid and reliable tool for assessing eating behaviour traits among French-Speaking Canadian children and adolescents. This questionnaire could represent an important assessment tool to better understand childhood obesity and help in the development of strategies and interventions aimed to prevent unhealthy eating behaviour traits and childhood obesity.

## Supplementary material

For supplementary material accompanying this paper visit https://doi.org/10.1017/S136898002100392X. click here to view supplementary material
