# Supplementary material for: Translation and validation of the Child Three-Factor Eating Questionnaire (CTFEQr17) in French-speaking Canadian children and adolescents
Source: Public Health Nutr. 2021 Sep 10;25(3):543–53. doi: 10.1017/S136898002100392X (PMC9991566; doi:10.1017/S136898002100392X)
Supplement: Supplementary file 1 [file S136898002100392Xsup001.docx]

**APPENDIX - CTFEQr17 questionnaire**

Note: Cognitive restraint comprises of items 1, 5 and 11. Uncontrolled eating comprises of items 3, 6, 8, 9, 12, 13, 15 and 17. Emotional eating comprises of items 2, 4, 7, 10, 14 and 16.

**Consignes/**Lis chaque énoncé et encercle l’affirmation qui correspond le mieux à ce que tu fais en général.

1. **Je mange des petites portions au repas pour m’aider à contrôler mon poids.** Totalement vrai (=4); Plutôt vrai (=3); Plutôt faux (=2); Totalement faux (=1).
2. **Je commence à manger quand je me sens inquiet.** Totalement vrai (=4); Plutôt vrai (=3); Plutôt faux (=2); Totalement faux (=1).
3. **Parfois, quand je commence à manger, il semble que je ne peux pas m’arrêter.** Totalement vrai (=4); Plutôt vrai (=3); Plutôt faux (=2); Totalement faux (=1).
4. **Quand je suis triste, je mange habituellement trop.** Totalement vrai (=4); Plutôt vrai (=3); Plutôt faux (=2); Totalement faux (=1).
5. **Je ne mange pas certains types d’aliments parce qu’ils peuvent me faire grossir.** Totalement vrai (=4); Plutôt vrai (=3); Plutôt faux (=2); Totalement faux (=1).
6. **Quand je suis à côté de quelqu'un qui mange, j’ai aussi envie de manger.** Totalement vrai (=4); Plutôt vrai (=3); Plutôt faux (=2); Totalement faux (=1).
7. **Quand je me sens en colère, j’ai besoin de manger.** Totalement vrai (=4); Plutôt vrai (=3); Plutôt faux (=2); Totalement faux (=1).
8. **J’ai souvent tellement faim que je sens que je pourrais manger beaucoup sans me sentir plein.** Totalement vrai (=4); Plutôt vrai (=3); Plutôt faux (=2); Totalement faux (=1).
9. **Quand j’ai faim, je sens que je dois manger tous les aliments de mon assiette sans m’arrêter.** Totalement vrai (=4); Plutôt vrai (=3); Plutôt faux (=2); Totalement faux (=1).
10. **Quand je me sens seul, je me console en mangeant.** Totalement vrai (=4); Plutôt vrai (=3); Plutôt faux (=2); Totalement faux (=1).
11. **Au moment du repas, je mange moins que je le veux pour ne plus prendre du poids.** Totalement vrai (=4); Plutôt vrai (=3); Plutôt faux (=2); Totalement faux (=1).
12. **Lorsque je sens ou vois mon aliment préféré, je trouve difficile de m’arrêter de le manger même si je sors de table.** Totalement vrai (=4); Plutôt vrai (=3); Plutôt faux (=2); Totalement faux (=1).
13. **J’ai toujours assez faim pour manger quelle que soit l’heure.** Totalement vrai (=4); Plutôt vrai (=3); Plutôt faux (=2); Totalement faux (=1).
14. **Si je me sens nerveux, je tente de me calmer en mangeant.** Totalement vrai (=4); Plutôt vrai (=3); Plutôt faux (=2); Totalement faux (=1).
15. **Quand je vois quelque chose qui a l'air délicieux, j’ai souvent si faim que je dois le manger tout de suite.** Totalement vrai (=4); Plutôt vrai (=3); Plutôt faux (=2); Totalement faux (=1).
16. **Quand je me sens vraiment bouleversé, je veux manger.** Totalement vrai (=4); Plutôt vrai (=3); Plutôt faux (=2); Totalement faux (=1).
17. **À quelle fréquence as-tu faim?** Seulement au moment des repas (=1); Quelques fois entre les repas (=2); Souvent entre les repas (=3); Presque toujours (=4).
